# High Density Linkage Map Construction and QTL Detection for Three Silique-Related Traits in *Orychophragmus violaceus* Derived *Brassica napus* Population

**DOI:** 10.3389/fpls.2017.01512

**Published:** 2017-09-06

**Authors:** Yi Yang, Yusen Shen, Shunda Li, Xianhong Ge, Zaiyun Li

**Affiliations:** National Key Laboratory of Crop Genetic Improvement, National Center of Oil Crop Improvement, College of Plant Science and Technology, Huazhong Agricultural University Wuhan, China

**Keywords:** *Brassica napus*, quantitative trait locus (QTL), single nucleotide polymorphism (SNP), silique-related trait, candidate gene

## Abstract

Seeds per silique (SS), seed weight (SW), and silique length (SL) are important determinant traits of seed yield potential in rapeseed (*Brassica napus* L.), and are controlled by naturally occurring quantitative trait loci (QTLs). Mapping QTLs to narrow chromosomal regions provides an effective means of characterizing the genetic basis of these complex traits. *Orychophragmus violaceus* is a crucifer with long siliques, many SS, and heavy seeds. A novel *B. napus* introgression line with many SS was previously selected from multiple crosses (*B. rapa* ssp. *chinesis* × *O. violaceus*) × *B. napus*. In present study, a doubled haploid (DH) population with 167 lines was established from a cross between the introgression line and a line with far fewer SS, in order to detect QTLs for silique-related traits. By screening with a *Brassica* 60K single nucleotide polymorphism (SNP) array, a high-density linkage map consisting of 1,153 bins and spanning a cumulative length of 2,209.1 cM was constructed, using 12,602 high-quality polymorphic SNPs in the DH population. The average recombination bin densities of the A and C subgenomes were 1.7 and 2.4 cM, respectively. 45 QTLs were identified for the three traits in all, which explained 4.0–34.4% of the total phenotypic variation; 20 of them were integrated into three unique QTLs by meta-analysis. These unique QTLs revealed a significant positive correlation between SS and SL and a significant negative correlation between SW and SS, and were mapped onto the linkage groups A05, C08, and C09. A trait-by-trait meta-analysis revealed eight, four, and seven consensus QTLs for SS, SW, and SL, respectively, and five major QTLs (*cqSS.A09b, cqSS.C09, cqSW.A05, cqSW.C09*, and *cqSL.C09*) were identified. Five, three, and four QTLs for SS, SW, and SL, respectively, might be novel QTLs because of the existence of alien genetic loci for these traits in the alien introgression. Thirty-eight candidate genes underlying nine QTLs for silique-related traits were identified.

## Introduction

Oilseed rape (*Brassica napus* L., genomes AACC, 2*n* = 38) is the second-leading source of both vegetable oil and meal worldwide (USDA, 2017). The rapid growth of the global human population requires significant improvements in crop yield. The seed yield of *B. napus* is a complex trait that is determined by seeds per silique (SS), siliques per plant (SP), and seed weight (SW) (Chen et al., 2007), which are typical quantitative traits. As significant negative correlations among silique-related traits have been reported, it is critical to balance these traits in breeding programs (Hall et al., 2006; Shi et al., 2015) by identifying the quantitative trait loci (QTLs) or genes responsible for each trait. Because the complex nature of the yield associated traits in *B. napus* hampers conventional breeding programs, QTL mapping using molecular markers would reveal the genetic status of both yield components and silique-related traits. QTLs related to yield components in *B. napus* have been detected in different mapping populations (Clarke and Simpson, 1978; Butruille et al., 1999; Hall et al., 2006; Qiu et al., 2006; Udall et al., 2006; Chen et al., 2007; Radoev et al., 2008; Shi et al., 2009, 2011, 2015; Basunanda et al., 2010; Fan et al., 2010; Wang and Guan, 2010; Zhang et al., 2010, 2012; Ding et al., 2012; Yang et al., 2012; Li N. et al., 2014; Qi et al., 2014; Luo et al., 2017). QTLs for SS, SW, and SL have been mapped onto most *B. napus* chromosomes, except for A03, A04, A06, A10, C05, and C08, and accounted for 2.7–32.1, 0.7–34.8, and 1.9–65.6% of the total phenotypic variation in each mapping population, respectively.

Most linkage QTL mapping studies and association analysis studies involving silique-related traits in rapeseed have mainly consisted of primary mapping results with rare follow-up studies, making investigations into the genetic and molecular mechanisms underlying SS, SW, and SL difficult. This situation is likely attributable to two facts. First, the complicated allopolyploid genomes causes problems in QTL mapping, such as inaccuracies resulted from rearrangement of homologous sequences in different chromosomes. Second, the mainly used the second generation of molecular markers like SSR and AFLP in previous studies are at a low density across the genome and consequently result in low mapping precision. To date, only two silique-related trait genes have been cloned in rapeseed. One is the major QTL for SS, *qSS.C9* (Zhang et al., 2012), which played a role in regulating the formation of functional female gametophytes (Li et al., 2015). Another is an auxin-response factor gene (*ARF18*) involved in both SL and SW QTLs (Liu et al., 2015). *ARF18* inhibited the activity of downstream auxin genes, which regulated silique wall development and determined SW via maternal regulation (Liu et al., 2015).

QTL analysis has been accelerated by recent innovations in bioinformatic techniques and sequencing methods, due to their efficient high-throughput genotyping using single nucleotide polymorphism (SNP) markers that create high-density genome-coverage marker systems for the investigation of complex traits (Thomson, 2014). The 60K Illumina Infinium™ SNP array for *B. napus*, can deliver high-throughput, gene-based, and low-cost genotype screening for mapping populations (Delourme et al., 2013; Clarke et al., 2016). This system is efficiently powerful to localize the QTLs to a narrow genomic interval and even supply defined markers within QTL that controls the trait of interest (Kumar et al., 2017). Twelve genes that underlie eight QTLs for SW have been revealed by comparative mapping between *Arabidopsis* and *Brassica* species, and a gene-specific marker for *BnAP2* was developed (Cai et al., 2012). Using a set of 2,795 SNP/bin markers, a genetic linkage map covering 1,832.9 cM was constructed, and a previous major QTL for seed color and acid detergent lignin was localized to a small genomic interval on chromosome A09 (Liu et al., 2013).

The relatively narrow genetic diversity of modern breeding materials restricts the further improvement of yield potential (Basunanda et al., 2010), and the development of novel germplasm from diploid progenitors or other wild species is an important way of widening genetic variations for rapeseed (Qian et al., 2005; Basunanda et al., 2007; Zou et al., 2010; Girke et al., 2012; Snowdon et al., 2015). *Orychophragmus violaceus* (2*n* = 24, OO) of the *Brassicaceae* family is cultivated as an ornamental plant in China and is characterized with long silique, high seed number per silique, and large seeds (Luo et al., 1994). A single sexual intergeneric hybrid between *B. rapa* ssp. chinesis (2*n* = 20, AA) as the female and *O. violaceus* as the pollen parent was a partially fertile mixoploid (2*n* = 23–42), and contained the *B. rapa* complement plus some additional *O. violaceus* chromosomes, due to partial chromosome elimination (Li and Heneen, 1999). After pedigrees of individual F2 plants from this partial hybrid were advanced to the 10th generation by selfing, many novel, highly productive lines were established, which exhibited not only a wide variety of phenotypes but also variable seed quality. These lines had 2*n* = 36–40, with 2*n* = 38 being the most frequent, but no intact *O. violaceus* chromosomes (Xu et al., 2007). These lines contained a variable number of chromosomes from the A subgenome and 18 chromosomes form the C subgenome (unpublished data), possibly because the progenies of the hybrid at certain generations were pollinated by *B. napus* plants that were growing nearby. Lines with 2*n* = 38 and a *B. napus*-like chromosome complement produced siliques with far more seeds than the *B. rapa* parent or common *B. napus* cultivars but comparable with those of *O. violaceus*.

In this study, a *B. napus* doubled haploid (DH) population was developed from a cross between a novel introgression line and a line with far fewer SS, and was genotyped using a *Brassica* 60K Illumina Infinium™ SNP array for the purpose of constructing a high-density genetic map. The map, coupled with phenotypic data obtained from five environments, was then used to conduct QTL analysis for SS, SL, and SW. Five major QTLs for the three silique-related traits were detected, which should be fine-mapped for the improvement of silique-related traits in breeding programs.

## Materials and methods

### Plant materials

A DH population with 167 lines was developed by microspore culture from the cross between female parent No. 1167 and male parent HZ396 (Figure S1). No. 1167 was one DH line that was generated from a novel *B. napus* introgression via multiple crosses (*B. rapa* ssp. *chinesis* × *O. violaceus*) × *B. napus* (Li and Heneen, 1999; Xu et al., 2007) and had 2*n* = 38 with stable cytological behavior. As *O. violaceus* had a high number of seeds per pod (~40), the introgression had ~35 seeds per pod, but line No. 1167 had relatively few seeds per pod likely due to the genetic segregation. HZ396 provided by Prof. Guangsheng Yang, Huazhong Agricultural University had very few SS, because the female gametophytes aborted (Zhang et al., 2010). This DH population was used for genetic linkage map construction and subsequent QTL analysis.

### Experimental design and trait measurement

The 167 DH lines and the two parents were grown in a randomized complete block with multiple replicates and environments in 2015 and 2016 (Wuhan, Hubei Province, three replicates; Ezhou, Hubei Province, two replicates; Kunming, Yunnan Province, two replicates; and Chengdu, Sichuan Province, one replicate). The Wuhan, Ezhou, and Chengdu sites were semi-winter-type rapeseed growing environments, while Kunming was a spring-type site. Each line was grown in a plot in two rows with 10–13 plants in each row with spacing of 30 × 20 cm. Field management was conducted under standard agricultural procedures. All plots in different trials were harvested at the same time after all lines matured.

Silique-related traits (SS, SL, and SW) were used for QTL mapping, and open-pollinated seeds and siliques from the middle of the main inflorescence were used to measure SW, SS, and SL for each mature plant. The average length (not including the beak) and seed number of 10 well-developed siliques were used to measure the SS and SL of each plant, and the average weight of one thousand fully developed open-pollinated dry seeds was measured for SW.

### Genotyping and linkage map construction

DNA of the DH population and the two parents was extracted from young leaf samples before flowering. The leaf tissues were frozen in liquid nitrogen before the DNA was isolated using a Plant Genomic DNA Kit (CWBIO Inc., Beijing, China), following the manufacturer's instructions. After fluorometrically quantifying the DNA concentration using Qubit® 2.0, the samples were diluted to 50–100 ng/μL in sterile distilled water. The quantified DNA samples were then used for SNP analysis. The DH population and their parents were genotyped using the *Brassica* 60K Illumina Infinium™ SNP array (Clarke et al., 2016), according to the manufacturer's instructions (https://www.illumina.com/techniques/microarrays.html). Allele calling was conducted by Illumina Genome Studio data analysis software V2010.1. All of the called SNPs were searched against the *B. napus* cv. “Darmor-*bzh*” reference genome (Chalhoub et al., 2014) using the basic local alignment search tool (BLAST, *E*-value ≤ 1E-20). Markers with allelic frequencies lower than 0.05 and those with 10% missing values or more were removed.

Genetic linkage analysis was performed using MSTMap (Wu et al., 2008), which efficiently handled ultra-dense datasets. All of the genotypic data from the DH population were analyzed, and the MSTMap parameters were as follows: the distance_function was kosambi, the cut-off *p*-value was 1E-18, and no_map_dist was 15.0. Markers with the same genotypes in corresponding individuals were merged into one bin, which contained these redundant markers.

### Statistical analysis and QTL mapping

The phenotypic characteristics of the parents were compared using Student's *t*-tests, and phenotypic correlations between pairs of traits were calculated using the Pearson correlation coefficient with the procedure CORR in SAS software (SAS Institute Inc, 2000). Heritability was calculated as $\;h^{2}\;=\;\sigma_{g}^{2}/\;\left(\sigma_{g}^{2}\;+\;\sigma_{ge}^{2}/n\;+\sigma_{e}^{2}/\text{nr}\right)$ where $\sigma_{g}^{2}$ is genetic variance, $\sigma_{ge}^{2}$ is the variance representing genotype by environment interactions, $\sigma_{e}^{2}$ is the error variance, *n* is the number of environments, and r is the number of replicates. Year-location combinations were treated as different environments. Components of variance ($\sigma_{g}^{2}$, $\sigma_{ge}^{2},$ and $\sigma_{e}^{2}$) were estimated using the mixed linear model (MIXED) procedure in SAS, with environment included as a random effect. Subsequently, an R script based on a linear model was used to obtain the best linear unbiased prediction (BLUP) of the multi-environment phenotypes for each accession (Merk et al., 2012). The BLUPs and individual environment data were then included as phenotypes in a linkage analysis.

QTL analysis was performed by the composite interval mapping method (Wang et al., 2011) in Windows QTL Cartographer V2.5 (http://statgen.ncsu.edu/qtlcart/WQTLCart.htm). The experiment-wise logarithm (base 10) of odds (LOD) threshold value was determined after 1,000 permutations at the 0.05 significance level (Churchill and Doerge, 1994). A LOD score corresponding to *p* = 0.05 (3.2–6.5) was used to identify significant QTLs. QTLs with low effect values and corresponding threshold values higher than 2.5 for each trait were used to identify suggestive QTLs that had relatively small effects. After deleting non-reproducible suggestive QTLs, significant QTLs and reproducible suggestive QTLs remained and named as identified QTL. Identified QTLs detected in the different environments were integrated into consensus QTLs, and subjected to a meta-analysis using BioMercator v4.2 (Arcade et al., 2004). Consensus QTLs were identified trait-by-trait, and in the first round of the meta-analysis, two types of consensus QTLs were identified: major QTLs (those with *R*^2^ ≥ 20%, or *R*^2^ ≥ 10% in more than one environments) and minor QTLs (other QTLs with minor effect). In the second round, QTLs for diverse traits with overlapping confidence intervals (CIs) were integrated into unique QTLs.

### Candidate genes identification

We download all sequences of annotated genes derived from the *B. napus* reference enome “Darmor-*bzh*” (Chalhoub et al., 2014), then these sequences were used to conduct BLAST against the genes of *Arabidopsis*, with the threshold of 1E-30 for *E*-value. The best hit of BLAST results for each *B. napus* gene was considered as the homologs to *Arabidopsis*. Next, the genes for three silique-related traits and other potential associated traits (including flowing time and maturity time) for *Arabidopsis* were downloaded in bulk from the website (http://www.arabidopsis.org/tools/bulk/sequences/index.jsp). All homologs associated with these traits in *B. napus* were selected, and subsequently the homologous genes within the CIs of corresponding QTLs were selected as putative candidate genes for the QTLs.

## Results

### Phenotypic variation among DH lines

The two parents (No. 1167 and HZ396) differed significantly in SS, SW, and SL in all four environments investigated (Table 1). The average SS of No. 1167 was ~3-fold higher than that of HZ396, while the average SW of HZ396 was nearly 1.5 times that of No. 1167. The average SL of No. 1167 was nearly 1.6-times that of HZ396.

**Table 1:** Descriptive statistics for the three silique-related traits in the two parents and the DH population.

			**Parents**				**DH population**	
**Year**	**Locations**	**Traits*^a^***	**No.1167 mean ±*SD^b^***	**HZ396 mean ±*SD^b^***	**Significance level**	**Range**	**Mean ±*SD^b^***	**CV(%)*^c^***
2015	Wuhan	SS	19.17 ± 3.33	2.75 ± 1.34	^**^	4.32–27.28	17.17 ± 5.88	34.25
		SW	3.79 ± 0.32	5.18 ± 0.79	^**^	2.44–6.84	3.61 ± 0.67	18.56
		SL	5.25 ± 0.61	3.06 ± 0.38	^**^	2.58–6.81	4.89 ± 0.80	16.36
2015	Ezhou	SS	18.71 ± 2.02	6.26 ± 1.44	^**^	6.20–30.78	19.37 ± 6.05	31.23
		SW	3.61 ± 0.40	5.63 ± 0.41	^**^	2.65–6.42	3.72 ± 0.74	19.89
		SL	5.44 ± 0.50	3.78 ± 0.34	^**^	3.29–8.19	5.07 ± 0.78	15.38
2015	Kunming	SS	18.91 ± 2.71	5.49 ± 2.03	^*^	3.42–32.02	18.57 ± 6.79	36.56
		SW	4.20 ± 0.22	6.44 ± 0.16	^*^	3.34–7.47	4.71 ± 0.84	17.83
		SL	5.02 ± 0.28	3.30 ± 0.38	^**^	2.44–6.65	4.53 ± 0.81	17.88
2015	Chengdu	SS		6.15 ± 0.65		4.17–28.62	18.14 ± 5.61	30.93
		SW		5.78 ± 0.59		2.58–5.75	3.76 ± 0.53	14.10
		SL		19.40 ± 3.23		2.82–6.23	4.72 ± 0.71	15.04

** *Significant at P < 0.01*,

* *Significant at P < 0.05.

a *SS, seeds per silique, SW one thousand weight (g), SL silique length (cm)*.

b *SD standard deviation*.

c *CV coefficient of variation*.

**Figure 1:**
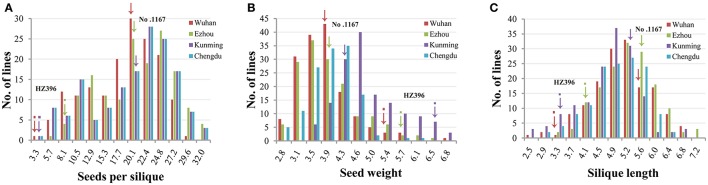
Phenotypic variation of the three traits in the DH populations. Panels **(A–C)** show the distribution of seeds per silique, seed weight, and silique length. The horizontal axis represents the trait value of seed number per pod, seed weight, and silique length. The vertical axis represents the number of individuals within the population. The different experiments are represented by different colors as indicated in the legend. Wuhan, Ezhou, Kunming, and Chengdu were the codes of the four experiments which have been in detail described in the first section of Methods.

Frequency distributions of all the traits measured in 167 DH lines were continuous and near the normal distribution in all off the environments (Figure 1), indicating a polygenic inheritance pattern. Regarding phenotypic variation in the entire mapping population, the traits had high coefficients of variation that ranged from 14 to 37%, indicating wide variation (Table 1). This observation was also verified by the results of an analysis of variance (ANOVA), which indicated highly significant differences among the DH lines (*p* < 0.001) in the three traits investigated (Table 2). Our results suggest that parental genotypes generated a huge amount of recombinants, which resulted in a wide range of genetic variation in the population and facilitated QTL mapping. In addition, the populations exhibited transgressive segregation of the three traits (Table 1), indicating that favorable alleles were mainly in the two parents. A two-way ANOVA of SS, SW, and SL across the environments revealed that the genotype of each DH (G), the growing environment (E), and a genotype-environment interaction (G × E) had significant effects on SS, SL, and SW (Table 2). However, the G × E effects were non-significant, because they were minor compared to the genotype and environment effects. Broad-sense heritability (*h*^2^) was also calculated for SS, SW, and SL (Table 2). SS and SW had high *h*^2^ -values of 90.3 and 90.6%, respectively, suggesting that they were stable and insensitive to environmental effects, which is generally consistent with the results of previous studies (Shi et al., 2009; Fan et al., 2010). The lower *h*^2^ -value of 79.3% for SL indicated that it was more sensitive and plastic to environmental conditions.

**Table 2:** Summary of all effects from two-way ANOVA of the traits in DH population.

**Trait**	**Variation**	***df***	**Mean Square**	***F*-value**	**Significance**	***h*^2^(%)*^a^***
SS	Genotype	160	177.15	19.20	^**^	90.32
	Environment	2	315.81	34.22	^**^	
	G × E	302	17.14	1.86	^**^	
	Error	374	9.23			
SW	Genotype	160	2.45	23.04	^**^	90.61
	Environment	2	101.97	960.88	^**^	
	G × E	300	0.23	2.13	^**^	
	Error	372	0.11			
SL	Genotype	160	3.47	6.10	^**^	79.25
	Environment	2	46.26	81.37	^**^	
	G × E	300	0.72	1.27	^*^	
	Error	392	0.57			

*For abbreviation, see Table 1*.

** *Significant at P < 0.001*,

* *Significant at P < 0.01*.

a *h^2^, broad-sense heritability*.

As expected, significant, positive correlations were found among SS, SL, and SW in each environment. The Pearson correlation analysis revealed that SS was significantly and positively correlated with SL, which is generally consistent with the results of previous studies (Zhang et al., 2012), but negatively correlated with SW. SW was significantly and negatively correlated with SL in almost all the environments (Table S1). All the traits studied were correlated with each other in most of the environments, indicating the co-dependence of the phenotypic responses.

### Construction of a high-resolution linkage map

**Figure 2:**
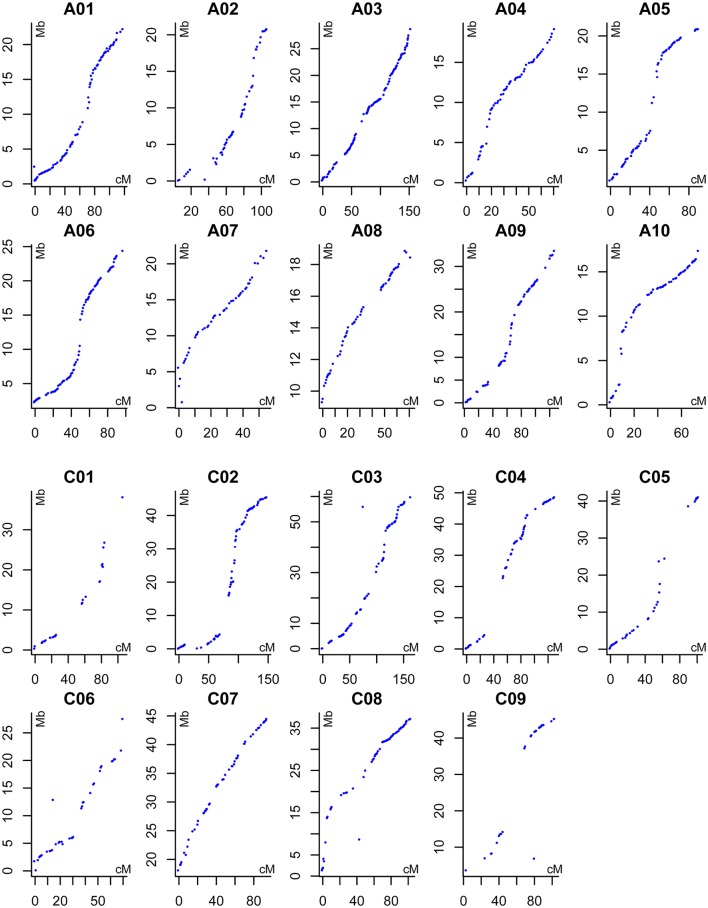
Alignments between the DH linkage map and the *B. napus* reference genome sequence. The X axis indicates the genetic position of each marker (cM), the Y axis indicates the physical position of reference sequence of each corresponding *B. napus* chromosomes (Mb).

The 167 DH lines and their parents were genotyped using 52,157 SNP loci, and 12,602 markers were found to be polymorphic using a series of quality filters. A high-density genetic map of 19 linkage groups (LGs), which were successfully assigned to the 19 chromosomes of the A (A01–A10) and C (C01–C09) subgenomes, was constructed using MSTMap (Table 3, Table S2, and Figure 2). We found that 6,617 and 5,985 markers were mapped onto the A and C subgenomes, respectively, confirming that the A subgenome had higher genetic diversity than the C subgenome. After clustering the redundant SNPs with same genotype, finally there were 1,153 bins (the region with no recombination in the population and considered as one marker with same genotype) left for the 19 LGs, and the bin number in the A subgenome was significantly higher than that in the C subgenome (720 vs. 433, *p* = 0.03). The lengths of the 19 LGs ranged from 70.2 cM (C06) to 220.6 cM (A09), with the sum and mean of 2,209.1 cM and 116.3 cM, respectively. The genetic distance between adjacent bins differed among LGs, and ranged from 1.1 to 3.7 cM, with an average interval of 1.7 and 2.4 cM for the A and C subgenomes, respectively (Table 3). In the process of data analyses, we found that three markers: Bn-A05-p18490530 (A05: 16,760,025..16,760,124), Bn-A07-p17639842 (A07: 19,527,996..19,528,095) and Bn-scaff_21003_1-p463835 (C08: 4,072,167..4,072,262) exhibited distorted segregation, with the segregation ratio of 96.3%, 92.1 and 88.6%, respectively. They all skewed toward the No. 1167 (female parent) and were not included in the linkage map.

**Table 3:** Summary statistics of the linkage map.

**Chr**	**Marker number**	**Bin number*^a^***	**Genetic distance**	**Marker density*^b^***	**Bin interval (cM)*^c^***	**Recombination frequency*^d^***
A01	626	89	118.52	7.0	1.3	5.4
A02	368	55	100.52	6.7	1.8	4.9
A03	1,019	113	153.19	9.0	1.4	5.4
A04	674	67	71.03	10.1	1.1	3.8
A05	646	62	124.17	10.4	2.0	6.3
A06	676	86	97.84	7.9	1.1	4.4
A07	787	66	156.86	11.9	2.4	8.9
A08	338	53	71.41	6.4	1.3	7.8
A09	764	70	220.59	10.9	3.2	6.6
A10	719	59	74.61	12.2	1.3	4.4
C01	1,215	24	106.47	50.6	4.4	2.8
C02	1,381	71	148.43	19.5	2.1	3.3
C03	817	78	164.92	10.5	2.1	2.7
C04	575	52	130.12	11.1	2.5	2.7
C05	303	34	101.84	8.9	3.0	2.5
C06	283	38	70.21	7.4	1.8	2.7
C07	344	49	94.34	7.0	1.9	3.6
C08	854	60	103.93	14.2	1.7	2.9
C09	213	27	100.08	7.9	3.7	2.3
A	6,617	720	1,188.74	9.2	1.7	5.8
C	5,985	433	1,020.33	15.2	2.4	2.8

a *The genetic region with no recombination in the population genetic location*.

b *The marker number per bin*.

c *The genetic interval for franked bin*.

d *The value calculated as the genetic length (cM) divided by the covered physical distance (Mb) of the corresponding linkage group*.

### QTL analysis of the three silique-related traits

**Figure 3:**
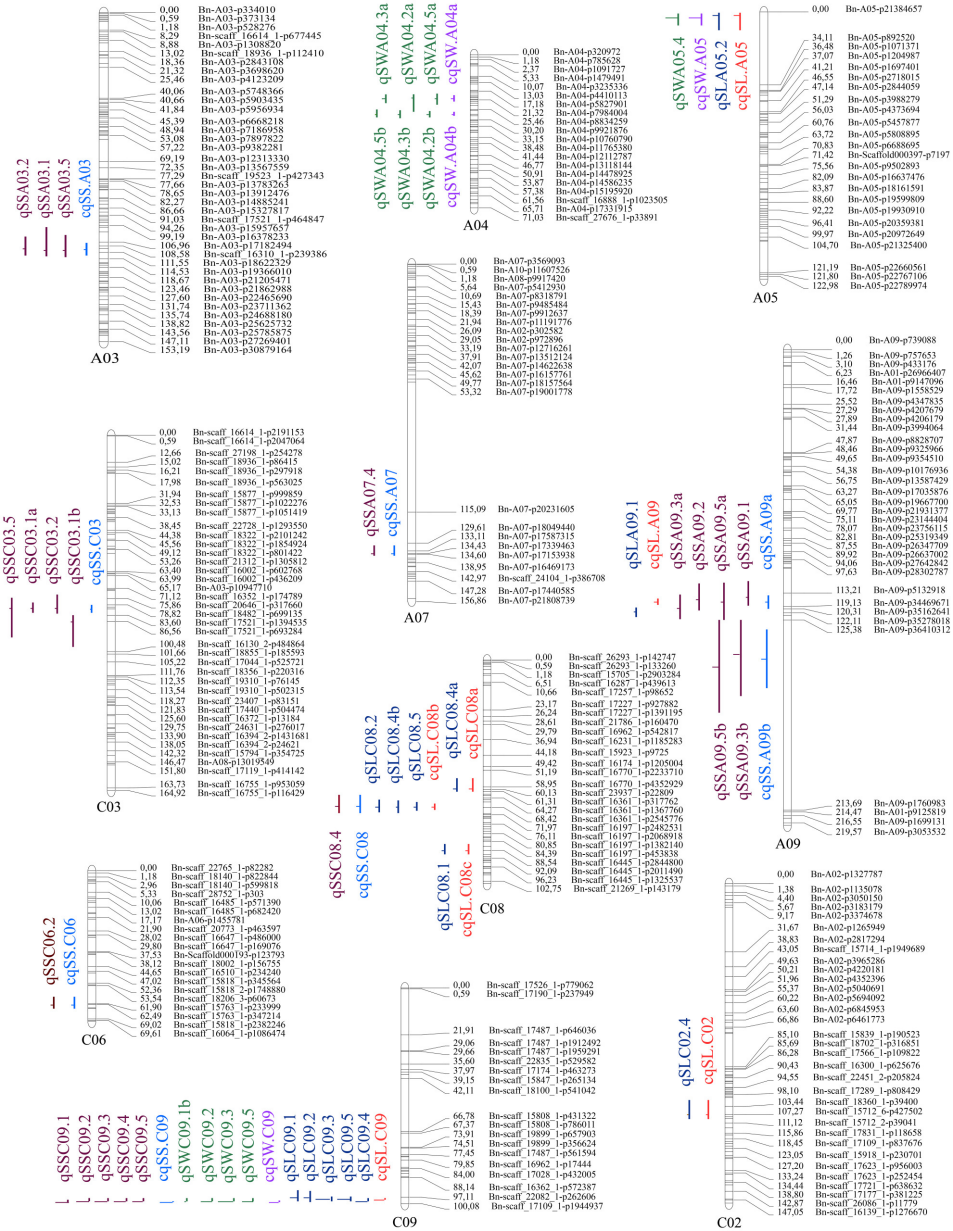
Genetic linkage map and QTL detection of the three silique-related traits in the DH population derived from No. 1167 × HZ396.

The genome-wide QTL analysis was performed separately for SS, SW, and SL using the 1,153 markers coupled with corresponding phenotypic data that were obtained from the DH population in five environments (four growing environments and the BLUP values across environments), and 52 QTLs were detected for three traits. After deleting seven non-reproducible, suggestive QTLs, 45 QTLs for SS, SL, and SW were detected in the five environments (Tables S3 and Figure 3). These QTLs were mapped onto 10 chromosomes (A03, A04, A05, A07, A09, C02, C03, C06, C08, and C09). The meta-analysis integrated QTLs that were located on the same LGs into consensus QTLs trait-by-trait. As a result, the 45 QTLs identified were integrated into 19 consensus QTLs (Table 4 and Figure 3), eight, four, and seven of them were detected for SS, SW, and SL, respectively. Some consensus QTLs, such as *cqSS.A09b, cqSS.C09, cqSWA05, cqSW.C09*, and *cqSL.C09*, had major effects, whereas others exhibited minor effects.

**Table 4:** Consensus QTLs for the silique-related traits investigated in the DH mapping population.

**Trait**	**Consensus QTL*^a^***	**LG**	**Position(cM) (cM)**	**CI *^b^***	***R*^2^ (%)**	**AE*^c^***	**QTL type*^d^***	**Environment**
SS	*cqSS.A03*	A03	110.3	107.3–113.4	4.11	−1.25	Minor	WH/EZ/BLUP values
	*cqSS.A07*	A07	134.4	130.8–135.9	6.16	−1.74	Minor	CD
	*cqSS.A09a*	A09	117.3	114.1–120.5	7.11	1.82	Minor	WH/EZ/KM/BLUP values
	*cqSS.A09b*	A09	143.8	130.1–157.5	17.96	2.74	Major	KM/BLUP values
	*cqSS.C03*	C03	81.1	79.0–83.1	3.55	−1.02	Minor	WH/EZ/BLUP values
	*cqSS.C06*	C06	62.5	58.6–64.3	6.61	−1.86	Minor	EZ
	*cqSS.C08*	C08	68.1	62.7–71.2	8.18	−1.74	Minor	CD
	*cqSS.C09*	C09	100.0	99.7–100.3	32.17	3.92	Major	WH/EZ/KM/CD/BLUP values
SW	*cqSW.A04a*	A04	20.6	19.1–22.2	6.37	0.18	Minor	EZ/KM/BLUP values
	*cqSW.A04b*	A04	27.8	26.8–28.8	5.59	0.17	Minor	EZ/KM/BLUP values
	*cqSW.A05*	A05	3.0	0.9–6.6	30.77	−0.94	Major	CD
	*cqSW.C09*	C09	99.7	99.3–100.1	25.92	−0.43	Major	WH/EZ/KM/BLUP values
SL	*cqSL.A05*	A05	2.0	0.4–8.6	14.25	0.84	Minor	EZ
	*cqSL.A09*	A09	122.1	120.3–124.8	8.40	0.26	Minor	WH
	*cqSL.C02*	C02	107.3	100.6–109.7	5.47	0.17	Minor	CD
	*cqSLC08a*	C08	60.1	54.8–61.3	8.46	−0.22	Minor	WH
	*cqSL.C08b*	C08	68.4	66.9–69.9	7.04	−0.2	Minor	EZ/CD/BLUP values
	*cqSL.C08c*	C08	88.5	85.5–90.3	7.78	−0.24	Minor	CD
	*cqSL.C09*	C09	99.5	98.9–100.0	26.54	0.46	Major	WH/EZ/KM/CD/BLUP values

a *According to consensus QTL nomenclature, cq is added to the trait name, and the LG number follows. When more than one QTLs were detected on one LG, a, b, or c was used to distinguish them*.

b *CI confidence interval (cM)*.

c *Additive effect: additivity indicate a positive additive means No. 1167 alleles increased phenotypic values*.

d *Major QTL: QTL with R^2^ ≥ 20% or with R^2^ ≥ 10% in more than one environments; minor QTL:other QTL with minor effect*.

For SS, 22 QTLs were detected in each environment, as well as the BLUP values across environments (Table S3). To avoid false-positive QTLs caused by a less-stringent LOD threshold, we deleted one non-reproducible, suggestive QTL. Consequently, 21 QTLs were identified for SS, which were on seven chromosomes (A03, A07, A09, C03, C06, C08, and C09). Individual QTLs explained 4.00–34.42% of the phenotypic variation in SS. These QTLs explained 54.15, 55.31, 58.13, 46.00, and 62.63% of the total phenotypic variation in Wuhan, Ezhou, Kunming, Chengdu, and the BLUP values, respectively. To examine whether the QTLs detected in the different environments were reproducible, we compared the CIs of QTLs mapped on the same chromosomes. Subsequently, the 21 QTLs were integrated into eight single consensus QTLs (Table 4). Of the eight consensus QTLs, two (*cqSS.A09b* and *cqSS.C09*) were identified in the mapping population and exhibited main effects and positive additive effects, indicating that the parent (No. 1167) contributed favorable alleles. Three minor QTLs (*cqSS.A03, cqSS.A09a*, and *cqSS.C03*) detected in multiple environments explained < 10.0% of the total phenotypic variation in each environment.

Thirteen QTLs for SW were detected in the different environments. After deleting two non-reproducible, suggestive QTLs, 11 QTLs were identified (Table S3), which were located on LG A04, A05, and C09, and explained 4.10–38.91% of the total phenotypic variation. If the CIs of these QTLs overlapped in different environments, they were integrated into a single consensus QTL for each trait. Four consensus QTLs were obtained (Table 4).

The consensus QTLs for SW explained 5.59–30.77% of the total phenotypic variation. The additive effects of the consensus QTLs for SW ranged from −0.94 to 0.18. Two major consensus QTLs (*cqSW.A05* and *cqSW.C09*) in the A05 and C09 LGs were detected. Because *cqSW.C09* was repeatedly detected in four environments and had the greatest phenotypic variation (*R*^2^ = 38.90%), it was treated as a major QTL. The effect of *cqSW.C09* was negative, meaning that the positive allele for SW was inherited from HZ396.

QTL analysis was also conducted for SL. Thirteen QTLs were identified in Wuhan, Ezhou, Kunming, Chengdu, and the BLUP values, which cumulatively explained 35.37, 33.48, 36.05, 53.94, and 39.33%, respectively, of the total phenotypic variation (Table S3), and then were integrated into seven consensus QTLs (Table 4). One consensus QTL (*cqSL.C09*) had positive additive effects, indicating that the parent (No. 1167) contributed the favorable allele.

As a previous study reported that correlated traits tended to share a higher proportion of QTLs than uncorrelated ones (Gardner and Latta, 2007), the correlations among SS, SW, and SL prompted us to examine whether the CIs of QTLs for SS, SW, and SL overlapped. The CIs of *cqSS.C09, cqSW.C09*, and *cqSL.C09* overlapped each other in the five environments (Table 4). The meta-analysis integrated these overlapping QTLs into one unique QTL, *uq.C09*, indicating that this QTL may have the had pleiotropic effect on SL, SW, and SL (Table 5). Two pairs of consensus QTLs (*cqSW.A05* and *cqSL.A05*, and *cqSS.C08* and *cqSL.C08b*) were integrated into two unique QTLs, which were renamed *uq.A05* and *uq.C08*, respectively. The unique QTL *uqC09* had different favorable allele contributions, *cqSS.C09* and *cqSL.C09* had positive additive effects, while the additive effects of *cqSW.C09* were negative at the same locus, which could explain the positive correlation between SS and SL and the negative correlation between SS and SW. The unique QTLs *uqA05* and *uqC08* that were mapped for SW and SL as well as SS and SL, respectively, exhibited the opposite trend of parental contributions.

**Table 5:** The list of pleiotropic unique (uq) QTLs obtained after meta-analysis in all environment using the DH population.

**Unique QTL**	**LG**	**Position (cM)**	**Traits correlated for pleiotrophic QTL**	**Associated markers**
*uq.A05*	A05	2.7	SW/SL	Bn-A05-p21384657
*uq.C08*	C08	68.4	SS/SL	Bn-scaff_16361_1-p2545776
*uq.C09*	C09	99.8	SS/SW/SL	Bn-scaff_17109_1-p1944937

*For abbreviation, see Table 1*.

### Alignment of present and previous QTLs

In order to facilitate the comparison and further utilization of QTLs for the *Brassica* researchers, we collected the results of the vast majority of QTL mapping studies conducted on *B. napus*. In total, 221, 489, and 78 QTLs for SS, SW, and SL, respectively, have been reported in *B. napus*. QTL positions from previous studies and the present study were compared based on the reference genome of *B. napus*, “Darmor-*bzh*” (Chalhoub et al., 2014). Finally, 177 QTLs for silique-related traits successfully mapped to the genome (Table S4 and Figure S2).

QTL *cqSS.A03* (present study) was identical to *qSN020* (Shi et al., 2009), and one previous QTL *qSN053* for SS (Shi et al., 2009) were identical to *cqSS.C08* (present study). Our *cqSS.C09* for SS was similar to *qSS.N19*, a major QTL on C09 identified using a DH population involving HZ396 used here (Zhang et al., 2011). QTL *cqSW.A04b* (present study) was identical to the QTL *qSW034* for SW (Shi et al., 2009). One QTL for SL on C08 identified by Udall et al. (2006) might be identical to one of the QTLs for SL here, and two QTLs for SL from Li N. et al. (2014) was possibly identical to our QTLs on A09 and C02. Seven previous QTLs were reproduced, and 12 QTLs were novel identified in the present study, five, three, and four of which were for SS, SW, and SL, respectively.

As previous studies suggest that various phenological traits such as flowering time and maturity time had certain effect on grain yield and its components (Kirkegaard et al., 2016), we investigated the QTLs associated with the traits mentioned above. Through conducting the alignment of identified QTLs with previously published studies on flowering time and maturity time, 9 and 11 QTLs overlapped with 9 and 10 consensus QTLs from this study, respectively. For instance, *qFT.A3-14 (FT), qMT.A3-4 (MT), qMT.A3-5 (MT), RF2qMT11 (MT)*, and *DHqMT12* (MT) overlapped with *cqSS.A03*; *qMT.A4-6* (MT) overlapped with *cqSW.A04a (MT)*; *qFT.A5-1* (FT), *RF2qFT43* (FT), *qFT093* (FT), *qMT.A5-3* (MT), and *RF2qMT13* (MT) overlapped with *uqA05*; *qMT.C8-5* (MT) overlapped with *uqC08*; *qFT.C9-3 (FT), dtf19.1 (FT)*, and, *qMT.C9-2* (MT) overlapped with *uq C09*. And others were listed in the Table S4.

### Prediction of silique-related genes in rapeseed

In order to predict candidate genes related to SS, SW, and SL, we identified *B. napus* genes that were homologous to *Arabidopsis thaliana* silique-related genes that were within the CIs of the QTLs. Based on the *B. napus* reference genome (Chalhoub et al., 2014), 27 homologous genes that controlled silique-related traits in *Arabidopsis* were within the CIs of nine consensus QTLs (Table S5 and Figure S2). Of these, *BnaA09g40790, BnaCnng45760, and BnaC09g45890* were identified for SS, which were homologous to the *Arabidopsis* genes *MOT1, TTG2*, and *SMG7*, respectively, and were within the CIs of *cqSS.A09a* and *cqSS.C09*. For SW, 28 genes were identified within the CIs of three consensus QTLs (*cqSW.A04a, cqSW.A05*, and *cqSW.C09)*, homologous to 23 *Arabidopsis* genes. For SL, eight genes were identified within the CIs of four consensus QTLs (*cqSL.A05, cqSL.A09, cqSLC08a*, and *cqSL.C09*), and were homologous to four *Arabidopsis* genes.

The homologs in *Arabidopsis* of candidate genes we identified in this study could be divided into several functional groups, including transcription factors, enzymes, protein structure units, phytohormone response factors, and transporters. Among these predicted genes, the most abundant were related to the transcription factors *AGL, ANT, FIE, IKU1, MINI3*, and *TTG2. AGL* encoded a MADS-domain protein that functioned as a transcription factor; *ANT* was required for the control of cell proliferation and encoded a putative transcriptional regulator similar to *AP2 (Manchado-Rojo et al*., *2014**)*; *FIS2* and *FIS3* encoded a Transducin/WD40 repeat-like superfamily transcription factor; and *IKU1, MINI3*, and *TTG2* were members of the *WRKY* transcription factor family. The enzymes were *AHK 1* (a histidine kinase 1), *CKI1* (a signal transduction histidine kinase), *KLU* (a cytochrome P450 monooxygenase) and *AHPs* (*AHP1, AHP2, AHP3, AHP4*, and *AHP5*), which encoded *A. thaliana* histidine phosphotransfer proteins that functioned as redundant positive regulators of cytokinin signaling. Protein structure units, such as *MSI1*, encoded a WD-40 repeat-containing protein that functioned as part of the *CAF1*-*FIE* complex. *ARF2* encoded an auxin response factor, and *MOT1* encoded a high-affinity molybdate transporter. We also found that the gene *AT1G26530* encoded a PIN domain-like family protein of unknown function.

Due to the availability of the *Arabidopsis* flowering time regulation network, 245 homologous genes of 103 flowering time-related genes in *Arabidopsis* were collected using the *in silico* mapping approach. Among them, 18, 21, 9 genes were mapped to the regions of QTLs for SS, SW and SL, respectively (Table S6). The vital genes underlying the QTLs for controlling flowering time were found, such as *BnaA07g25310* (*TSF*) in the confidence interval of *cqSS. A07*. Nearby the peak of *cqSS.A09a* and *cqSLC08a*, there existed *BnaA09g47030* (*DDF1*) and *BnaC08g25840* (*SMZ*), respectively. *BnaA07g24950* (*ATMBD9*) fell into the confidence interval of *cqSS.A07*, and *BnaA05g20150* (*FIE*) and *BnaA05g26370* (*PIE1*) were in the confidence interval of *uqA05*.

## Discussion

### Linkage map construction and silique-related-trait QTL detection

DH lines are used for silique-related QTL analysis because multiple locations can be used due to their stable genotypes, and interactions between silique-related QTLs and the environment can also be investigated. In this study, using a novel *B. napus* introgression that was derived from multiple crosses (*B. rapa* ssp. *chinesis* × *O. violaceus*) × *B. napus* and had many SS, we developed a DH population that exhibited great variation in all the traits investigated, and was ideal for genetic map construction and QTL detection (Figure 1). The molecular map developed in this study covered a total of 2,209.1 cM with an average interval of 1.9 cM between marker loci (Table 3), indicating high-density markers for possible applications in gene discovery studies. The good collinearity between the DH linkage map and the *B. napus* reference genome sequence confirmed the high quality of the present linkage map (Figure 2), which would guide the fine-mapping and map-based cloning of target genes underlying the QTLs by comparative mapping of the present linkage map and the physical map. Of the 45 QTLs identified for the three silique-related traits, 13 were novel and some had major effects, revealing the possible existence of alien genetic loci.

### Novel QTLs for SS, SW, and SL

Of silique-related trait QTLs from previous linkage-based QTL mapping studies, a total of 177 QTLs mapped to the genome (Table S4). Among them, 54 were identified for SS (Shi et al., 2009; Luo et al., 2017), 122 for SW (Butruille et al., 1999; Udall et al., 2006; Shi et al., 2009; Luo et al., 2017), and one for SL(Udall et al., 2006), which were on 16 (A01–A05, A07–A10, C01, C03-C06, C08, and C09), 19 (A01–A10 and C01–C10) and one (C08) LGs, respectively (Table S4).

Fifty-two QTLs for the three traits were identified in our DH population. After deleting seven non-reproducible, suggestive QTLs in the first round of the meta-analysis, 45 QTLs were integrated into 19 consensus QTLs. Most of these QTLs had a moderate effect (*R*^2^ < 20%), and five (*cqSS.A09b, cqSS.C09, cqSW.A05, cqSW.C09*, and *cqSL.C09*) could be considered major QTLs. Of these, ten were repeatedly detected, and the other nine were environment-specific (Table 4). These QTLs were distributed on 10 LGs (A03, A04, A05, A07, A09, C02, C03, C06, C08, and C09). To accurately identify the positional relationships of the QTLs from our and previous studies, a comparative QTL analysis was performed based on the physical map of *B. napus* (Chalhoub et al., 2014). Eight SS, four SW, and seven SL consensus QTLs were identified in present study, and three (*cqSS.A03, cqSS.C08* and *cqSS.C09*), one (*cqSW.A04-2*) and three (*cqSL.A09, cqSL.C02* and *cqSL.C08*), respectively, were found by previous studies. The QTLs in present study, *cqSS.A03* and *cqSS.C08*, probably corresponded to previous *qSN020* and *qSN053*, respectively, and were located around the common markers CNU276 and CB10504 (Shi et al., 2009). A major QTL (*qSS.N19*) for SS (Zhang et al., 2011) was similar to our *cqSS.C09*. Furthermore, Li et al. (2015) identified the *BnaC9.SMG7b* gene underlying the *qSS.N19* QTL by map-based cloning. The consensus QTLs *cqSW.A04b* and *cqSL.C08a* were overlapped with *qSW034* and *ln18.5* QTLs, respectively (Udall et al., 2006; Shi et al., 2009), and two QTLs for SL identified by Li N. et al. (2014) might be identical to our QTLs on A09 and C02. More importantly, QTLs for SL on A05 and C09 were detected for the first time. Our other 12 consensus QTLs (*cqSS.A07, cqSS.A09a, cqSS.A09b, cqSS.C03, cqSS.C06, cqSW.A04a, cqSW.A05, cqSW.C09, cqSL.A05, cqSL.C08b, cqSL.C08c, and cqSL.C09*) should be novel. Of these novel consensus QTLs, *cqSS.A09b, cqSW.A05, cqSW.C09*, and *cqSL.C09* were major QTLs detected in this study, and would be important targets for map-based cloning. Unfortunately, the DH line No. 1167 which was used as a parent to develop the mapping population had few SS than the original introgression. Otherwise, more major QTLs for these traits may be identifiable. Nevertheless, our results show that all three traits are controlled by many loci that mainly have small effects.

### Co-localization of QTLs for silique-related traits

From the second round of the meta-analysis revealing significant co-localization of QTLs, three unique QTLs were identified and located on the LGs A05, C08, and C09, and all had pleiotropic effects. Such QTLs should be separated from QTLs that affected more than one trait because of genetic linkage. These unique, pleiotropic QTLs confirmed the result that a significant, positive correlation existed between SS and SL and a significant, negative correlation between SW and SS (Zhang et al., 2011). As pleiotropic genes had large genetic effects on trait mapping (Wang and Guan, 2010), the QTLs on A05 and C09 showed larger genetic effects and higher LOD scores than other QTLs. The significant co-localizations indicated that the silique-related traits were dependent upon each other, and pleiotropic QTLs were the main genetic factors for the silique-related traits in our DH population. The loci of pleiotropic QTLs contained many tightly linked, trait-specific genes, or genes that affected multiple traits (Hall et al., 2006). The meta-analysis allowed us to localize the genomic region for QTLs, which would facilitate studies into molecular cloning and QTL functioning by comparing the *B. napus* and *A. thaliana* genomes. Interestingly, genes for SW and cell expansion effects in *A. thaliana*, such as *MINI3, TTG2*, and *MSI1* (Table S5; Cai et al., 2012, 2016; Wang et al., 2016), their homologous gene in *Brassica napus* were assigned to the CI of *uq.A05*, which controlled SW and SL. For SW, *TTG2* increased seed size by increasing cell expansion, *DA1* increased SW and organ size, and *IKU1* and *MINI3* promoted endosperm growth in *Arabidopsis*. As rapeseed siliques played a critical role in seed yield, the candidate genes for silique-related QTLs need to be confirmed.

As we know, grain yield and silique-related traits were complex agronomic traits and were influenced by the environmental factors, such as the soil fertility, photoperiod and temperature. The previous studies indicated that the flowering time and mature time contributed to the seed yield of oilseed rape (Kirkegaard et al., 2016). Yield showed negative correlation with flowering time and lines that flowered earlier had higher yield than late-flowering lines (Raman et al., 2016). Therefore, it was relevant for investigating the flowering associated genes within the confidence intervals of the QTLs for three silique-related traits. Multiple homologs of flowering associated genes of *Arabidopsis*, such as *TSF, SOC1, ATDDF1, ATMBD9*, and *FIE*, were identified in some QTLs intervals, inferring that these genes might affect the development of the silique and the seeds with the form of pleiotropy or influence the seeds filling through controlling the time of flowering. Two genes: *FT* and *SOC1* influenced flowering time on various pathways, which were often referred to as the floral integrator genes (Nelson et al., 2016). *TSF* encoded a floral inducer as a homolog of *FT* which played overlapping roles in the promotion of flowering. *SOC1* controlled flowering and was required for *CO* to promote flowering. It acted downstream of *FT. DDF1* encoded an AP2 domain transcription factor that could repress flowering. In rapeseed, *BnAP2*, an *APETALA2* (*AP2*)-like gene, affected seed size, structure and development (Yan et al., 2012). *BnaA07g24950* (*ATMBD9*) was involved in the modification of the *FLC* chromatin acetylation state to affect *FLC* expression. The *FIE* encoded a transcriptional regulation protein and formed part of a large protein complex that could include VRN2 and VIN3, while the complex had a role in establishing *FLC* repression during vernalization. The mutations in *PIE1* resulted in suppression of *FLC*-mediated delay of flowering and caused early flowering in non-inductive photoperiods independently of *FLC*. However, now we could not find the strong evidence for the effect of these flowering genes on the identified QTLs here because the confidence intervals of QTLs still had several centimorgans, especially the relatively lower density markers in C subgenome. It was suggested that the flowering- and silique-related genes were probably just tightly linked in the certain physical interval, like the linked genes *Chalk5* and *GS5* in rice (Li et al., 2011; Li Y. et al., 2014). So the fine mapping of these QTLs may give us more information about their relationships.

In conclusion, 45 individual QTLs were detected for three silique traits (SS, SW and SL) in the *O. violaceus* derived *B. napus* population. Among 19 consensus QTLs, 12 (5, 3, 4 for SS, SW, and SL, respectively) were novel, likely due to the introgression of alien genetic loci. Thirty-eight candidate genes underlying nine QTLs for three traits were identified. Our study provides new insights into the genetic control of these important traits for seed yield, and highlights the role of special germplasm in genetic analyses.

## Author contributions

ZL and XG conceived and designed the study. YY, YS, and SL performed the phenotyping measurement. YY and YS performed the data analysis. YY wrote the manuscript and all authors reviewed and edited the manuscript.

### Conflict of interest statement

The authors declare that the research was conducted in the absence of any commercial or financial relationships that could be construed as a potential conflict of interest.
