# Supplementary figures and images for: High Density Linkage Map Construction and QTL Detection for Three Silique-Related Traits in Orychophragmus violaceus Derived Brassica napus Population
Source: Front Plant Sci. 2017 Sep 6;8:1512. doi: 10.3389/fpls.2017.01512 (PMC5592274; doi:10.3389/fpls.2017.01512)

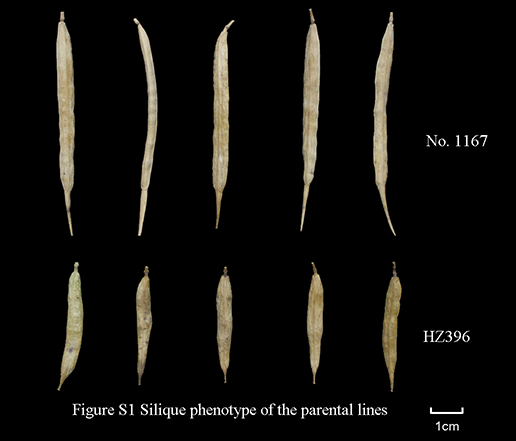

Supplement: Supplementary file 7 [file Image1.TIF]

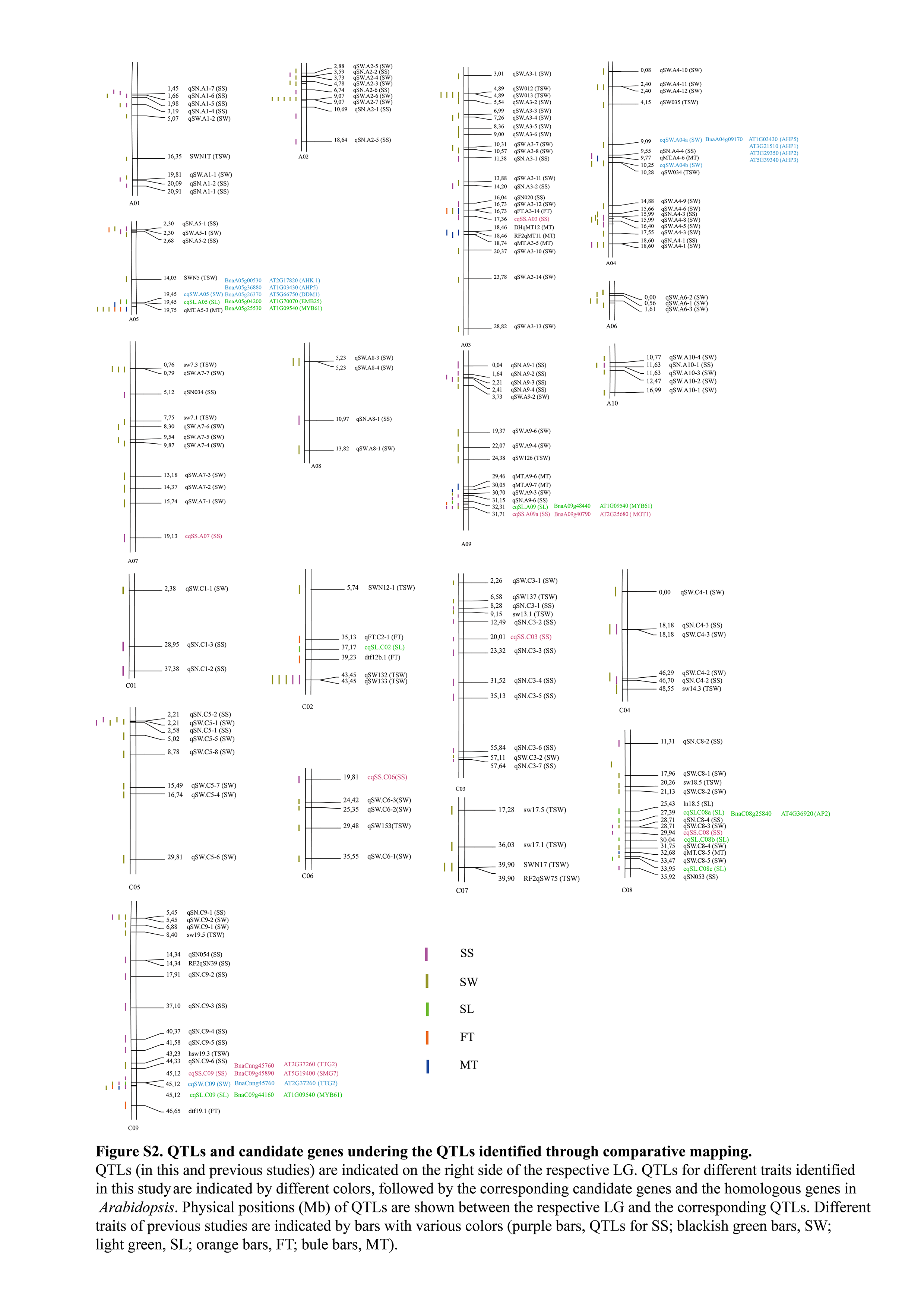

Supplement: Supplementary file 8 [file Image2.TIF]
